# Milk Yield and Milk Fatty Acids from Crossbred F1 Dairy Cows Fed on Tropical Grasses and Supplemented with Different Levels of Concentrate

**DOI:** 10.3390/ani12192570

**Published:** 2022-09-26

**Authors:** Laura Haydeé Vallejo Hernández, Arni Xochitemol Hernández, Epigmenio Castillo Gallegos, Manuel Gonzalez-Ronquillo, Einar Vargas-Bello-Pérez, Luis Corona

**Affiliations:** 1 Department of Animal Science, Autonomous University of Chapingo, Texcoco 56230, Mexico; 2 Graduate Programs in Animal Health and Production, College of Veterinary Medicine and Animal Science, National Autonomous University of Mexico, Mexico City 04510, Mexico; 3 Center for Teaching, Research and Extension in Tropical Livestock, College of Veterinary Medicine and Animal Science, National Autonomous University of Mexico, Mexico City 04510, Mexico; 4 Department of Animal Nutrition, College of Veterinary Medicine and Animal Science, Autonomous University of Mexico State, Toluca 50000, Mexico; 5 Department of Animal Sciences, School of Agriculture, Policy and Development, University of Reading, P.O. Box 237, Earley Gate, Reading RG6 6EU, UK; 6 Department of Animal Nutrition and Biochemistry, College of Veterinary Medicine and Animal Science, National Autonomous University of Mexico, Mexico City 04510, Mexico

**Keywords:** conjugated linoleic acid, grazing, rumenic acid, vaccenic acid, dairy products

## Abstract

**Simple Summary:**

The objective of this study was to determine milk fatty acids (FA) from crossbred F1 dairy cows fed on tropical grasses and supplemented with different levels of concentrate. Milk yield and milk composition were not affected while very mild effects were found in milk fatty acids. Overall, this study shows that adding up to 450 g/kg of concentrate to crossbred F1 dairy cows fed on tropical grasses does not have negative effects on milk yield and milk quality. Therefore, under these production conditions, farmers can avoid the use of concentrate, rely on tropical grasses, and reduce feeding costs.

**Abstract:**

The objective of this study was to determine milk fatty acids from crossbred F1 dairy cows fed on tropical grasses and supplemented with different levels of concentrate. Twelve dairy cows (50% Holstein × 50% Brahman) with 60 days of lactation grazing tropical grasses were assigned to a Switchback design, with three periods of 15 days with different concentrate levels: 0, 150, 300 and 450 g /kg. Milk samples were obtained on the last five days of each experimental period. Milk yield and milk composition were not affected. Cows fed with 300 g/kg of concentrate had higher contents of C15:0 (*p* = 0.004), C22:0 (*p* = 0.031), and C24:0 (*p* = 0.013). C17:1 cis9 was higher (*p* = 0.039) with 150 g/kg and lowest with 450 g/kg. C18:1 cis9 was higher (*p* = 0.042) with 150 g/kg. C18:2n6trans was higher (*p* = 0.05) with 300 g/kg and lower (*p* = 0.018) with 450 g/kg. This study shows that adding up to 450 g/kg of concentrate to crossbred F1 dairy cows fed on tropical grasses does not have negative effects on milk yield and milk quality. Therefore, under these production conditions, farmers can rely on tropical grasses and reduce feeding costs.

## 1. Introduction

In the tropical regions of Mexico, a first cross-generation (F1) from temperate dairy breeds from *Bos taurus* and tropical local cattle breeds from *Bos indicus* was carried out to obtain an animal that is able to cope with temperature and relative humidity [1]. These animals are commonly fed on tropical grasses [2]. Today, consumers are aware of saturated FA in milk and they prefer to avoid its intake [3]. However, in milk from grazing cows, the presence of unsaturated FA could be higher compared to cows fed under total mixed rations [4]. 

The need for improved milk yields in F1 dairy cows has led farmers to increase the amount of dietary concentrate but its increase can lead to milk fat depression [5], digestive disorders such as acidosis [6] or increases in milk saturated FA [3]. The response to different levels of nutrient supply in dairy cows depends on various factors, such as genetics, stage of lactation, type of feeding, feed quality and climate [7]. Although many research efforts have been carried out on milk fatty acid profile and its modulation by dietary means, information on milk FA from crossbred F1 dairy cows reared under tropical conditions remains a field of study that deserves attention. Therefore, this short communication had the objective of determining milk fatty acids from crossbred F1 dairy cows fed on tropical grasses and supplemented with different levels of concentrate. The hypothesis of this study was that increasing dietary concentrate would lead to an increased milk yield as well as an increase in the contents of saturated FA in milk.

## 2. Materials and Methods

### 2.1. Experimental Site

The study was carried out at the Center for Teaching, Research and Extension in Tropical Livestock (C.E.I.E.G.T.) of the National Autonomous University of Mexico (U.N.A.M.) (20°04’ N and 97°03’ W), at an altitude that varies between 99 and 123 m above sea level, with an average annual temperature of 24.5 °C and average annual rainfall of 1991 ± 352 mm.

### 2.2. Animals and Diets

Animal care and procedures were carried out according to the guidelines of the animal care committee of the Universidad Nacional Autónoma de México (project code IT202120). Twelve F1 dairy cows (50% Holstein × 50% Brahman) with 60 days of lactation and average production of 6 kg of milk per day were assigned to a switchback design, with three periods of 15 days with different concentrate levels at 0, 150, 300 and 450 g of DM concentrate/kg of daily milk production over 6 kg/d. 

The amount of commercial concentrate (ABATEZ^®^, Mexico) was supplied according to the treatment during milking time, from 7:30 to 10:00 h, and was made off of 43.3% corn grain; 36.8% sorghum grain, 12.9% soybean meal, 6.0% molasses, and 1% mineral and vitamins premix. The concentrate had 89% dry matter, 18.3% crude protein, 37.4% neutral detergent fiber, 26.2% acid detergent fiber and 9%acid detergent lignin while offered meadows had 20% dry matter, 10.9% crude protein, 72.4% neutral detergent fiber, 38.5% acid detergent fiber and 5.6% acid detergent lignin. 

Animals grazed over 29 paddocks that on average had 1.62 ± 0.705 ha. The experimental cows grazed together with the milking herd that comprised 67 cows with an average weight of 490 ± 49 kg. The stocking rate was equivalent to 1.56 animal units (AU = 450 kg of LW), with a total grazing area of 46.87 ha. The grass-based pastures were composed of 28% local, mixed native pastures *Paspalum notatum*, *P. conjugatum*, *Axonopus affinis*, *Desmodium triflorum* and 72% exotic, mixed introduced species *Brachiaria Humidicola*, *Cynodon niemfluensis*, *B. brizantha* Toledo, *B. decumbens*, *Digitaria decumbens*, and *B. brizanta* Marandu. Cattle had an ample provision of tree shade and fresh water, both supplied right at pasture. 

The experimental cows were the last to go into milking to give them time to consume all concentrate. Milking occurred once a day between 07:30 and 10:00 h and milk production was registered as kg/cow/day. The milking parlor was a combination of parallel and herringbone types (“parabone”), capable of milking eight cows at a time.

### 2.3. Milk Yield and Milk Fatty Acid Profile

In the last five days of each experimental period, milk production was recorded and pooled samples of 200 mL from each cow were taken for fatty acid analysis. 

Milk fat separation was carried out using a non-solvent method and the transesterification of FA as reported previously [8]. C13:0 was the internal standard and was used for quantification. Fatty acids were analyzed using a gas chromatograph Perkin Elmer Autosystem XL (Perkin Elmer, Shelton, CT, USA), equipped with a flame ionization detector (FID). A Supelco SP2560 capillary column of 100 m × 0.25 mm × 0.2µm film thickness was used. Fatty acids were annotated using a 37-component FAME MIX (47885-U; Sigma-Aldrich, Inc., St. Louis, MO, USA), rumenic acid (O-5507; Sigma-Aldrich, Inc., St. Louis, MO, USA), and a trans-vaccenic acid (V1131; Sigma-Aldrich, Inc., St. Louis, MO, USA) as external standards. Before analyzing samples, the GC-FID system was calibrated using the external standards and all calibration curves were linear with correlation coefficients above 0.99. Calibration curves for FA were constructed based on 4 standard dilutions and applying linear regression analysis on the concentration ratio (μg/mL of compound per μg/mL of internal standard) and peak area ratio (area of compound/area of internal standard). Four concentrations (100, 1000, 5000 and 10,000 μg/mL of total FAME) were prepared by diluting the FAME MIX with hexane. Triplicate analysis was performed at each concentration level. Retail whole milk samples were used for quality control and were run before, during and after GC analysis to assess system suitability.

### 2.4. Statistical Analysis

Milk fatty acids were analyzed using a switchback design for four treatments with three blocks and three periods [9] and using the SAS PROC MIXED procedure [10] (SAS, 1990) according to the following model:

Yijkl = μ + δ_l_ + β_i(l)_ + α_j_ + γ_k_ + αγ_jk_ +τ_j_ + ε_ijkl_ (1)

The model included treatment, period and sequence as fixed effects and cows as random effects where Y_ijkl_ is the measured response variable; i—cow; j—treatment; k the period; and l the sequence. Then, μ is an overall mean, δl is the fixed effect due to the sequence of l, β _i(l)_ is a random effect due to the item nesting within the sequence l, α_j_ is a fixed effect due to treatment j, γ_k_ is a fixed effect due to period k, αγ_jk_ is a fixed interaction effect due to treatment j and period k, and εi_jkl_ is the random error.

Milk fatty acid means were subjected to orthogonal polynomial trend analysis [11]. Effects were considered significant if they were less than *p* < 0.05, using Tukey’s test for means comparison.

## 3. Results and Discussion

Overall production performance and milk composition are reported in a companion paper [12]. Briefly, dry matter intake (ranging from 12.7 to 13.4 kg/d) and milk yield (ranging from 7.35 to 8.75 kg/d) were not affected by dietary concentrate levels. In milk, fat (ranged from 3.50 to 3.79 g/100 g), protein (ranged from 2.38 to 2.51 g/100 g) and lactose (ranged from 3.50 to 3.79 g/100 g) were similar between treatments. Similarly, in grazing cows, Lawrence [13] and Dale [14] reported no changes in milk yield and milk composition in grazing Holstein cows fed with different dietary concentrate levels. One explanation for our findings could be that the concentrate allocation strategy has little effect on milk production, especially when the same quantity of concentrate is offered and when forage allowance is supplied ad libitum, as is the case in this study, due to the contribution of nutrients by foraging and the use of body reserves. Yet, scarce data are available on the response of grazing crossbred F1 dairy cows to different concentrate allocation strategies. Taken together, if milk yield and quality cannot be improved, our findings on production are relevant for farmers as a reduction in the use of concentrates represents a fall in feeding costs which signifies around 70% of the cost of milk production.

In contrast to productive traits, dietary levels of concentrate had effects on the milk fatty acid profile. Cows fed with 300 g/kg of concentrate had higher contents of C15:0 (*p* = 0.004), C22:0 (*p* = 0.031), and C24:0 (*p* = 0.013) compared with the rest of the treatments (Table 1). 

These differences are very mild and do not seem to be biologically significant; however, it is possible that feeding crossbred F1 dairy cows with 300 g/kg of concentrate was not enough to affect the rumen biohydrogenation process where the main end products are C16:0 and C18:0. Extensive biohydrogenation of dietary unsaturated fatty acids in the rumen is one of the reasons why enriching ruminant products with unsaturated fatty acids is a very difficult task [5]. Although intake of saturated fatty acids is related to negative effects on human health, there are data showing that not all saturated fatty acids exert cardiometabolic effects on human health and short- (from 2 to 5 carbon atoms) and medium- (from 6 to 12 carbon atoms) chain fatty acids could be beneficial [15]. 

Contents of C17:1 cis9 were highest (*p* = 0.039) with 150 g/kg of concentrate and lowest with 450 g/kg (Table 2). In ruminants, this fatty acid is produced from C17:0 by Δ9-desaturase in the mammary gland and its content in milk fat could be a reflection of forage-to-concentrate rations and protein supply as they affect rumen biohydrogenation of dietary polyunsaturated fatty acids [16].

Oleic acid (C18:1 cis9) was higher (*p* = 0.042) with 150 g/kg but lower with 0 and 450 g/kg. In humans, intake of oleic acid was reported to be a strategy for managing metabolic disorders such as obesity, insulin resistance, dyslipidemia, and high blood cholesterol [17].

Contents of C18:2n6trans were higher (*p* = 0.05) with 300 g/kg of concentrate and lower (*p* = 0.018) with 450 g/kg, while C20:2 was higher with 450 g/kg (Table 3). Previously, Patel et al. (2013) reported a decrease in the contents of C18:2n6 as the proportion of dietary forage increased in cows fed on diets based on grass silage. This change was accompanied by an increase in C18:2 cis9, trans11, but in this study, just numerical differences were found, and the highest contents were detected with 300 g/kg of concentrate. Vaccenic acid (C18:1 trans11) is a C18:1 isomer and is a precursor for C18:2 cis9, trans11, and their contents are desirable in milk for human consumption. Rumenic acid is associated with many beneficial effects on humans, for example, C18:2 cis9, trans11 induces beneficial changes in immune modulators associated with sub-clinical inflammation in overweight adults [18] as well as being related to gut integrity and inflammation in obese adults [19].

Previously, Bargo et al. [20] reported that increasing concentrate levels in grazing cow diets result in increased contents of saturated fatty acids and reduced unsaturated fatty acids in milk. However, in this study, the total contents of the main fatty acid groups (Table 4) were not affected by dietary concentrate included at different levels. These results agree with those from productive traits. This study shows that adding up to 450 g/kg of concentrate to crossbred F1 dairy cows fed on tropical grasses does not have negative effects on milk yield and milk quality. Therefore, under these production conditions, farmers can avoid the use of concentrate, rely on tropical grasses, and reduce feeding costs. 

When interpreting results from milk fatty acid profiles, it is important to point to the fact that in this study, C13:0 was used as an internal standard and this fatty acid is of rumen microbial origin and can be found in trace amounts in milk. From an analytical perspective, future studies should consider using a different internal standard for this type of sample.

## 4. Conclusions

In general, this study shows that adding up to 450 g/kg of concentrate to crossbred F1 dairy cows fed on tropical grasses does not have negative effects on milk yield and milk quality, as shown by the lack of differences in milk yield and minor changes in milk fatty acids. Overall, since milk yield and quality were not improved, concentrate supplementation is not needed at least under the conditions of the present study.

## Figures and Tables

**Table 1:** Saturated fatty acids (g/100 g of total fatty acids) in milk from crossbred F1 dairy cows fed on tropical grasses and supplemented with different levels of concentrate (0, 150, 300 and 450 g of DM concentrate/kg of daily milk production).

Fatty Acid	Treatment				SEM	*p*-Value		
	0	150	300	450		Treatment	Linear	Quadratic
C15:0	1.46 ^b^	1.42 ^b^	1.53 ^a^	1.30 ^c^	0.034	0.004	0.051	0.027
C16:0	28.0	27.3	26.2	29.8	0.613	0.104	0.362	0.040
C17:0	1.05 ^a^	1.05 ^a^	1.04 ^a^	0.92 ^b^	0.018	0.008	0.004	0.043
C18:0	12.6	11.6	12.7	11.9	0.395	0.613	0.691	0.920
C20:0	0.23	0.21	0.24	0.22	0.007	0.335	0.901	0.850
C22:0	0.11 ^b^	0.13 ^a^	0.13 ^a^	0.11 ^b^	0.005	0.031	0.700	0.004
C23:0	0.07	0.11	0.07	0.07	0.011	0.317	0.573	0.312
C24:0	0.10 ^c^	0.11 ^b^	0.12 ^a^	0.09 ^d^	0.003	0.013	0.132	0.008

SEM = Standard error of the mean. Means in the same row with different superscripts differ (*p* < 0.05).

**Table 2:** Monounsaturated fatty acids (g/100 g of total fatty acids) in milk from crossbred F1 dairy cows fed on tropical grasses and supplemented with different levels of concentrate (0, 150, 300 and 450 g of DM concentrate/kg of daily milk production).

Fatty Acid	Treatment				SEM	*p*-Value		
	0	150	300	450		Treatment	Linear	Quadratic
C14:1 cis9	0.89	1.10	1.01	1.04	0.047	0.254	0.299	0.231
C16:1 cis9	2.06	2.27	1.98	2.06	0.092	0.508	0.642	0.658
C17:1 cis9	0.37 ^b^	0.47 ^a^	0.37 ^b^	0.33 ^c^	0.019	0.039	0.136	0.048
C18:1 trans11	3.29	3.05	3.54	2.82	0.138	0.065	0.291	0.205
C18:1 cis9	20.6 ^c^	24.3 ^a^	23.0 ^b^	20.5 ^c^	0.652	0.042	0.699	0.006
C20:1	0.18	0.22	0.22	0.18	0.011	0.081	0.889	0.011
C22:1n9	0.006	0.009	0.009	0.0001	0.003	0.143	0.166	0.065

SEM = Standard error of the mean; Means in the same row with different superscripts differ (*p* < 0.05).

**Table 3:** Polyunsaturated fatty acids (g/100 g of total fatty acids) in milk from crossbred F1 dairy cows fed on tropical grasses and supplemented with different levels of concentrate (0, 150, 300 and 450 g of DM concentrate/kg of daily milk production).

Fatty Acid	Treatment				SEM	*p*-Value		
	0	150	300	450		Treatment	Linear	Quadratic
C18:2n6trans	0.14 ^b^	0.14 ^b^	0.17 ^a^	0.10 ^c^	0.010	0.005	0.067	0.007
C18:2n6cis	0.91	1.06	0.92	1.13	0.037	0.242	0.207	0.693
C18:3n3	0.40	0.41	0.33	0.35	0.018	0.113	0.069	0.843
C18:2 cis9, trans11	1.15	1.31	1.41	0.98	0.086	0.071	0.432	0.017
C20:2	0.04 ^c^	0.04 ^c^	0.05 ^b^	0.06 ^a^	0.008	0.018	0.040	0.023
C20:3n6	0.04	0.03	0.04	0.02	0.003	0.333	0.179	0.423
C20:4n6	0.07	0.03	0.05	0.05	0.008	0.437	0.650	0.313
C22:2	0.02	0.02	0.02	0.03	0.005	0.882	0.533	0.652
C20:5n3	0.05	0.04	0.04	0.03	0.002	0.052	0.008	0.731

SEM = Standard error of the mean; Means in the same row with different superscripts differ (*p* < 0.05).

**Table 4:** Total fatty acid (g/100 g of total fatty acids) groups in milk from crossbred F1 dairy cows fed on tropical grasses and supplemented with different levels of concentrate (0, 150, 300 and 450 g of DM concentrate/kg of daily milk production).

Fatty Acid	Treatment				SEM	*p*-Value		
	0	150	300	450		Treatment	Linear	Quadratic
Saturated fatty acids	64.7	60.7	62.0	65.9	0.930	0.060	0.434	0.010
Monounsaturated fatty acids	27.6	31.3	30.1	27.2	0.767	0.071	0.654	0.012
Polyunsaturated fatty acids	2.88	3.10	3.02	2.67	0.118	0.351	0.388	0.116
Others	4.59	4.53	4.68	4.04	0.094	0.703	0.635	0.542

SEM = Standard error of the mean; Means in the same row with different superscripts differ (*p* < 0.05).

## Data Availability

The raw data supporting the conclusions of this article will be made available by the authors, without undue reservation.
